# Peer Review of “Google Trends as a Predictive Tool for COVID-19 Vaccinations in Italy: Retrospective Infodemiological Analysis”

**DOI:** 10.2196/38724

**Published:** 2022-04-19

**Authors:** Zubair Shah

**Affiliations:** 1 College of Science and Engineering Hamad Bin Khalifa University Ar-Rayyan Qatar

**Keywords:** COVID-19, epidemiology, Google Trends, infodemiology, infoveillance, Italy, public health, SARS-CoV-2, vaccinations, vaccines, social media analysis, social media

*This is a peer-review report submitted for the paper “Google Trends as a Predictive Tool for COVID-19 Vaccinations in Italy: Retrospective Infodemiological Analysis.”*

## Round 1 Review

### General Comments

The paper [1] uses Google Trends (GT) to identify correlations between search queries and vaccinations. GT has been used previously by others for similar and other problems. The paper is well written. The Methods section can be improved. The Results section has a good explanation.

### Specific Comments

#### Major Comments

1. The novelty of the paper is limited.
2. The Introduction is short and can be extended to include more relevant studies.
3. The Methods section needs more details. For instance, how GT works, especially when keywords are two words “vaccine reservation.” Does it search for all queries that include both words vaccine and reservation or vaccine OR reservation, or does it search for an exact match (“vaccine reservation”)? More search terms can be included, such as synonyms of reservation like an appointment or booking. Additionally, how was data normalized? What is lag week?

## Abbreviations

GT: Google Trends

## References

[1] Rovetta A (2022). Google Trends as a predictive tool for COVID-19 vaccinations in Italy: a retrospective infodemiological analysis. JMIRx Med.

